# Neurodevelopmental Diagnoses Before, During, and After the COVID-19 Pandemic

**DOI:** 10.1001/jamanetworkopen.2026.5683

**Published:** 2026-04-08

**Authors:** Sloane J. Freeman, Rosane Nisenbaum, Michael D. Sgro

**Affiliations:** 1 Women and Children’s Health Program, St Michael’s Hospital, Unity Health Toronto, Toronto, Ontario, Canada; 2 MAP Centre for Urban Health Solutions, Li Ka Shing Knowledge Institute, St Michael’s Hospital, Unity Health Toronto, Toronto, Ontario, Canada; 3 Faculty of Medicine, University of Toronto, Toronto, Ontario, Canada; 4 Strategic Partnerships in Health Excellence, Research, and Engagement, MAP Centre for Urban Health Solutions, Li Ka Shing Knowledge Institute, St Michael’s Hospital, Unity Health Toronto, Toronto, Ontario, Canada; 5 Division of Biostatistics, Dalla Lana School of Public Health, University of Toronto, Toronto, Ontario, Canada

## Abstract

**Importance:**

The COVID-19 pandemic and associated lockdowns resulted in changes to the way children learned, developed, and accessed health care.

**Objective:**

To evaluate changes in rates and rate trends of new neurodevelopmental diagnoses for children in Ontario, Canada, before, during, and after the COVID-19 pandemic.

**Design, Setting, and Participants:**

This population-based retrospective cohort study used administrative data from ICES to assess new neurodevelopmental diagnoses from March 14, 2015, to December 31, 2024, among 291 896 children aged 6 years or younger in Ontario.

**Exposures:**

The COVID-19 pandemic period, defined as March 14, 2020, to November 30, 2022, and the postpandemic period, defined as December 1, 2022, to December 31, 2024.

**Main Outcomes and Measures:**

Monthly rate of new neurodevelopmental diagnoses per 1000 child-months, defined as the total number of children with a neurodevelopmental diagnosis divided by the total number of child-months. Interrupted time series analysis using data before, during, and after the pandemic was performed to evaluate changes in rate levels or slopes between study periods.

**Results:**

A total of 291 896 children received a neurodevelopmental diagnosis during the study period. Of 1 481 844 children at risk, 130 418 received a diagnosis in the prepandemic period (mean [SD] age, 2.5 [1.6] years; 79 573 boys [61.0%]); of 1 115 791 children at risk, 86 383 received a diagnosis during the pandemic period (mean [SD] age, 2.4 [1.6] years; 52 943 boys [61.3%]); and of 1 014 792 children at risk, 75 095 received a diagnosis in the postpandemic period (mean [SD] age, 2.7 [1.6] years; 45 030 boys [60.0%]). In the prepandemic period, rates of neurodevelopmental diagnoses increased monthly (slope = 0.04; 95% CI, 0.03-0.05; *P* < .001). In the first month of the pandemic, rates of neurodevelopmental diagnoses decreased by 0.91 per 1000 child-months (95% CI, −1.56 to −0.25 per 1000 child-months; *P* = .01) compared with the expected rate in the absence of the pandemic. Rates recovered and remained stable, with no statistically significant differences in slopes between the prepandemic period and either during or after the pandemic.

**Conclusions and Relevance:**

This population-based cohort study showed no significant differences in rates of new neurodevelopmental diagnoses among children aged 6 years or younger from prepandemic rates in the pandemic and postpandemic periods. The rapid deployment of virtual care may have helped maintain health care access, facilitating diagnoses during the pandemic and postpandemic periods.

## Key Points

**Question:**

How was the COVID-19 pandemic associated with rates of new neurodevelopmental diagnoses among children 6 years or younger in Ontario, Canada?

**Findings:**

This population-based cohort study of 291 896 children showed no significant differences in new neurodevelopmental diagnoses from prepandemic rates either during or after the pandemic.

**Meaning:**

This study suggests that the rapid deployment of virtual care delivery in Ontario may have been associated with the maintenance of health care access during the pandemic and after the pandemic.

## Teaser

This cohort study evaluates changes in rates and rate trends of new neurodevelopmental diagnoses for children aged 6 years or younger in Ontario, Canada, before, during, and after the COVID-19 pandemic.

## Introduction

The incidence of neurodevelopmental disorders is increasing and represents a major public health concern in the US and Canada.^1,2^ Neurodevelopmental disorders are characterized by impairments in cognitive, behavioral, social, and academic functioning.^3,4^ Prior to the COVID-19 pandemic, 24% of publicly insured and 11% of privately insured children in the US were expected to receive at least 1 neurodevelopmental diagnosis by 8 years of age.^3^ Data from the National Longitudinal Survey of Children and Youth in Canada showed that more than 9% of children between 4 and 11 years of age have a neurodisability.^5^

The COVID-19 pandemic and associated lockdowns resulted in changes to the way children learned, developed, and accessed health care.^6^ Schools shifted to virtual learning and daycares closed for extended periods. Social distancing and restrictions on public spaces such as parks and playgrounds limited opportunities for social connection, conversation, and play. In addition, public health measures aimed at limiting the spread of the virus dictated abrupt changes to health care delivery. In Ontario, Canada, outpatient care either operated at reduced capacity or closed completely to in-person care after March 12, 2020, for the remainder of the year and throughout parts of 2021.^7,8^ Most pediatric developmental and mental health care shifted to virtual delivery.^9^

Although prenatal exposure to SARS-CoV-2 infection has not been consistently associated with neurodevelopmental sequelae, emerging evidence suggests that such exposure, as well as pandemic-related changes to early life experiences, may be associated with neurodevelopmental delays. Considering this evidence as well as the limitations in pediatric developmental assessments during the pandemic, we sought to evaluate the rates of new neurodevelopmental diagnoses for children in Ontario before, during, and after the COVID-19 pandemic. We hypothesized that neurodevelopmental diagnoses would decrease during the pandemic period and increase during the postpandemic period.

## Methods

### Study Design and Setting

We conducted a population-based retrospective cohort study of new neurodevelopmental diagnoses among children aged 6 years or younger in Ontario, Canada, from March 14, 2015, to December 31, 2024. We used administrative data from ICES (formerly called The Institute for Clinical Evaluative Sciences), an independent research institute, the legal status of which under Ontario’s health information privacy law allows it to collect and analyze health care and demographic data without individual patient consent for health system evaluation and improvement.^10^ Databases accessed through ICES included Statistics Canada and the Ontario Registered Persons Database (RPDB), which provided sociodemographic data. Age and sex were obtained from the RPDB, while neighborhood income quintile and rurality were derived from RPDB postal code information and linked with census data from Statistics Canada using the Postal Code Conversion File. Rurality was defined using standard ICES classifications based on community size and distance to urban centers. We included neighborhood income quintiles because socioeconomic status is associated with neurodevelopmental diagnoses.^3^ Neighborhood income quintiles were defined as the nearest census-based neighborhood income quintile within a census metropolitan area or a census agglomeration, with 1 being the lowest quintile and 5 being the highest.^11^ The physician billings database (Ontario Health Insurance Plan [OHIP] database) was accessed through ICES to identify physician diagnostic billing codes for each health care visit.^12^ The list of physician diagnostic billing codes for all neurodevelopmental diagnoses, including autism and developmental delays, can be found in eTable 1 in Supplement 1. Any physician (specialist or primary care) can use these diagnostic billing codes. The Unity Health Toronto research ethics board approved this study, and a waiver of participant consent was granted because only aggregated data were obtained from ICES. This study followed the Strengthening the Reporting of Observational Studies in Epidemiology (STROBE) reporting guideline.^13^

### New Developmental Diagnosis and Eligible Population

A new neurodevelopmental diagnosis was defined as the presence of 2 new diagnostic billing codes at least 30 days apart for a neurodevelopmental problem at 2 consecutive health care visits to a physician. The index visit was defined as the first visit at least 30 days after the initial visit. All children aged 6 years or younger residing in Ontario between March 14, 2015, and December 31, 2024, with an OHIP number (health insurance) and who did not have a new neurodevelopmental diagnosis prior to the start of the study period were eligible. The data from these children are entered into the RPDB on issuance of an OHIP number; therefore, eligibility was defined using RPDB registration rather than health care service use.

### Exposures

Our exposures were the pandemic period, defined as March 14, 2020, to November 30, 2022, and the postpandemic period, defined as December 1, 2022, to December 31, 2024. We selected March 14, 2020, as the start of the pandemic period because the Ontario Ministry of Health implemented virtual care billing codes on this date to facilitate care delivery during government-mandated lockdowns and restrictions at the onset of the pandemic. Because there is no official definition of the postpandemic period in Ontario, we defined the postpandemic period as beginning December 1, 2022, reflecting the lifting of COVID-19 public health restrictions and normalization of health care services.

### Primary Outcome

Our primary outcome measure was the monthly rate of a new neurodevelopmental diagnosis per 1000 child-months, defined as the total number of children with a neurodevelopmental diagnosis divided by the total number of child-months. We defined 1 month as a period of 30 consecutive calendar days, independent of health care encounters. Person-months for each child (ie, child-months) were calculated separately for each study period. Follow-up began at the later of the child’s birth date or the start of the period of interest and ended at the earliest of the date of death, loss of OHIP eligibility, second diagnosis, end of the study period, or the child’s sixth birthday. If a diagnosis occurred within a given month, the child contributed less than 1 full month of person-time when one of these events occurred during that month. Children who met the eligibility criteria were reassessed on a monthly basis.

### Statistical Analysis

Descriptive statistics (mean [SD] values median [IQR] values, and counts and percentages) and graphs were used to summarize the data and illustrate trends in the prepandemic, pandemic, and postpandemic periods. Interrupted time series analysis (ITSA) was performed to compare changes in monthly rates between the prepandemic, pandemic, and postpandemic periods.

We anchored the interval from March 14 to April 12, 2020, to represent the first month of the pandemic period. When assembling the data needed for the ITSA, we observed that the first prepandemic interval from March 14 to April 9, 2015, included only 27 days, and the last postpandemic interval from December 18 to 31, 2024, included only 14 days. To allow 30 or more days between initial and index visits (to count diagnoses) and keep equally spaced time points of exactly 30 days (for the ITSA), we excluded the first interval (which did not contribute any new diagnoses) and the last interval of the series from our analyses. Therefore, we redefined the periods for the ITSA data as the prepandemic period, from April 10, 2015, to March 13, 2020; the pandemic period, from March 14, 2020, to November 28, 2022; and the postpandemic period, from November 29, 2022, to December 17, 2024. The prepandemic period included 60 months, the pandemic period included 33 months, and the postpandemic period included 25 months.

We used the segmented regression model proposed by Wagner et al^14^ and the parameterization model proposed by Huitema and McKean.^15^ After determining that the distribution of rates did not substantially deviate from a symmetric distribution, Stata, version 15 (StataCorp LLC)^16^ command itsa estimated ordinary least-squares regression coefficients with Newey-West standard errors, which handle autocorrelation between the monthly rates over time and heteroscedasticity. We used the command actest to perform the Cumby-Huizinga tests for autocorrelation to determine the specific lag order up to 12. Sensitivity ITSA analyses were conducted to evaluate linear trends within each period. To this end, we added (1) an interruption to the prepandemic series at 13 months to assess the influence of outliers at the beginning of the period and (2) another interruption on the month after the first-year anniversary of the pandemic to assess the initial changes after the pandemic (eTable 2 in Supplement 1). Additional exploratory stratified ITSA was conducted by sex (eTable 3 and eFigure 2 in Supplement 1). A multiple-group ITSA model tested the difference in slopes within each period between girls and boys. Statistical significance was defined as a 2-sided *P* < .05.

## Results

A total of 291 896 children received a new neurodevelopmental diagnosis during the study period; of 1 481 844 children at risk, 130 418 received a diagnosis in the prepandemic period (mean [SD] age, 2.5 [1.6] years; 79 573 boys [61.0%] and 50 845 girls [39.0%]); of 1 115 791 children at risk, 86 383 received a diagnosis during the pandemic period (mean [SD] age, 2.4 [1.6] years; 52 943 boys [61.3%] and 33 440 girls [38.7%]); and of 1 014 792 children at risk, 75 095 received a diagnosis in the postpandemic period (mean [SD] age, 2.7 [1.6] years; 45 030 boys [60.0%] and 30 065 girls [40.0%]) (Table 1).^11^ Sociodemographic characteristics at index visits are presented in Table 1.^11^ See eTable 1 in Supplement 1 for the frequencies of neurodevelopmental diagnostic codes in each period. The total number of eligible children, total child-months, and overall rates of new neurodevelopmental diagnoses per 1000 child-months, stratified by sex and by age group for each period, are shown in Table 2. Male children demonstrated higher rates of new neurodevelopmental diagnoses across all study periods. Infants aged 12 months or younger displayed the lowest rates of neurodevelopmental diagnoses across all study periods. Most diagnoses were made within 12 months of the initial visit (81.5% [106 339 of 130 418] in the prepandemic period, 74.1% [64 019 of 86 383] in the pandemic period, and 73.5% [55 178 of 75 095] in the postpandemic period). The median number of months from initial visit to diagnosis was 4.4 months (IQR, 2.4-9.3 months) for the prepandemic period, which increased in the pandemic period to 5.2 months (IQR, 2.6-12.4 months) and the postpandemic period to 5.4 months (IQR, 2.8-12.7 months).

**Table 1.** Sociodemographic Characteristics at the Time of New Neurodevelopmental Diagnosis

Characteristic	Children, No. (%)		
	Prepandemic period (March 14, 2015, to March 13, 2020)	Pandemic period (March 14, 2020, to November 30, 2022)	Postpandemic period (December 1, 2022, to December 31, 2024)
No.	130 418	86 383	75 095
Age at first diagnosis, mean (SD), y	2.5 (1.6)	2.4 (1.6)	2.7 (1.6)
Sex, No. (%)			
Female	50 845 (39.0)	33 440 (38.7)	30 065 (40.0)
Male	79 573 (61.0)	52 943 (61.3)	45 030 (60.0)
Neighborhood income quintile, No. (%)^a^			
Unidentified	554 (0.4)	229 (0.3)	181 (0.2)
Lowest	26 290 (20.2)	17 066 (19.8)	14 435 (19.2)
Second	23 091 (17.7)	15 711 (18.2)	13 588 (18.1)
Third	24 186 (18.5)	16 942 (19.6)	14 605 (19.4)
Fourth	25 017 (19.2)	16 733 (19.4)	14 328 (19.1)
Highest	21 481 (16.5)	14 046 (16.3)	12 086 (16.1)
Rural region^b^	9799 (7.5)	5656 (6.5)	5872 (7.8)

^a^ Neighborhood income quintiles are defined as the nearest census-based neighborhood income quintile within a census metropolitan area or a census agglomeration, with 1 being the lowest quintile and 5 being the highest.^11^

^b^ Rural area defined as community size of fewer than 10 000.

**Table 2.** New Neurodevelopmental Diagnoses by Study Time Period Stratified by Sex and Age Group

Characteristic	Prepandemic period (March 14, 2015, to March 13, 2020)	Pandemic period (March 14, 2020, to November 30, 2022)	Postpandemic period (December 1, 2022, to December 31, 2024)
**All children**			
No. of children with a new neurodevelopmental diagnosis	130 418	86 383	75 095
Total No. of children at risk	1 481 844	1 115 791	1 014 792
Total No. of child-months	46 318 494.4	24 186 458.0	18 172 130.4
Rate of new neurodevelopmental diagnosis (per 1000 child-months)	2.8	3.6	4.1
**Female children**			
No. of children with a new neurodevelopmental diagnosis	50 845	33 440	30 065
Total No. of children at risk	728 147	551 724	503 785
Total No. of child-months	23 006 260.5	12 060 350.4	9 099 395.4
Rate of new neurodevelopmental diagnosis (per 1000 child-months)	2.2	2.8	3.3
**Male children**			
No. of children with a new neurodevelopmental diagnosis	79 573	52 943	45 030
Total No. of children at risk	753 697	564 067	511 007
Total No. of child-months	23 312 233.9	12 126 107.6	9 072 333.8
Rate of new neurodevelopmental diagnosis (per 1000 child-months)	3.4	4.4	5.0
**Aged ≤12 mo**			
No. of children with a new neurodevelopmental diagnosis	14 289	9993	7071
Total No. of children at risk	837 986	515 170	426 571
Total No. of child-months	8 446 094.0	4 532 652.0	3 488 718.0
Rate of new neurodevelopmental diagnosis (per 1000 child-months)	1.7	2.2	2.0
**Aged >12 to 36 mo**			
No. of children with a new neurodevelopmental diagnosis	53 841	38 865	29 424
Total No. of children at risk	940 237	622 967	525 421
Total No. of child-months	15 797 312.9	8 356 730.8	6 365 546.0
Rate of new neurodevelopmental diagnosis (per 1000 child-months)	3.4	4.7	4.6
**Aged >36 mo to 6 y**			
No. of children with a new neurodevelopmental diagnosis	62 288	37 525	38 600
Total No. of children at risk	1 000 936	670 276	579 669
Total No. of child-months	22 074 915.4	11 296 944.1	8 317 364.2
Rate of new neurodevelopmental diagnosis (per 1000 child-months)	2.8	3.3	4.6

The ITSA model accounted for autocorrelation up to lag 4 (*P* = .008 determined by the Cumby-Huizinga test). During the prepandemic period, rates of new neurodevelopmental diagnoses appeared to increase every month (slope = 0.04; 95% CI, 0.03-0.05; *P* < .001) (Table 3). In the first month after the pandemic was declared, there was a decrease in diagnosis rates of 0.91 per 1000 child-months (95% CI, −1.56 to −0.25 per 1000 child-months; *P* = .01) compared with what would have happened in the absence of the pandemic. Monthly rates did not significantly change during the pandemic period (slope = 0.03; 95% CI, −0.002 to 0.06; *P* = .07) but increased during the postpandemic period (slope = 0.03; 95% CI, 0.02-0.04; *P* < .001). There were no statistically significant differences in rate slopes between the prepandemic period and either the pandemic or postpandemic period. The Figure displays the observed rates, model estimated trends, and counterfactual line with 95% CIs. In addition, sex-stratified analyses showed similar rate trends across the study periods (eTable 3 and eFigure 2 in Supplement 1). There were no statistically significant differences in slopes between boys and girls for the prepandemic, pandemic, and postpandemic periods.

**Table 3.** Interrupted Time Series Analysis of Monthly Rates of Neurodevelopmental Diagnosis Per 1000 Child-Months

Parameter	Estimate (95% CI)	*P* value
Prepandemic period		
Rate level at beginning of the prepandemic period	1.73 (1.23 to 2.23)	<.001
Prepandemic slope	0.04 (0.03 to 0.05)	<.001
Pandemic period		
Change in rate level that occurred immediately after start of pandemic period vs counterfactual prepandemic period	−0.91 (−1.56 to −0.25)	.01
Change in rate trends or difference between pandemic and prepandemic slopes	−0.01 (−0.05 to 0.02)	.48
Pandemic slope	0.03 (−0.002 to 0.06)	.07
Postpandemic period		
Change in rate level that occurred immediately after start of postpandemic period vs counterfactual pandemic period	−0.21 (−0.60 to 0.18)	.29
Change in rate trend or difference between postpandemic and pandemic slopes	0.003 (−0.03 to 0.04)	.84
Change in rate trend or difference between postpandemic and prepandemic slopes	−0.01 (−0.03 to 0.01)	.38
Postpandemic slope	0.03 (0.02 to 0.04)	<.001

**Figure.**
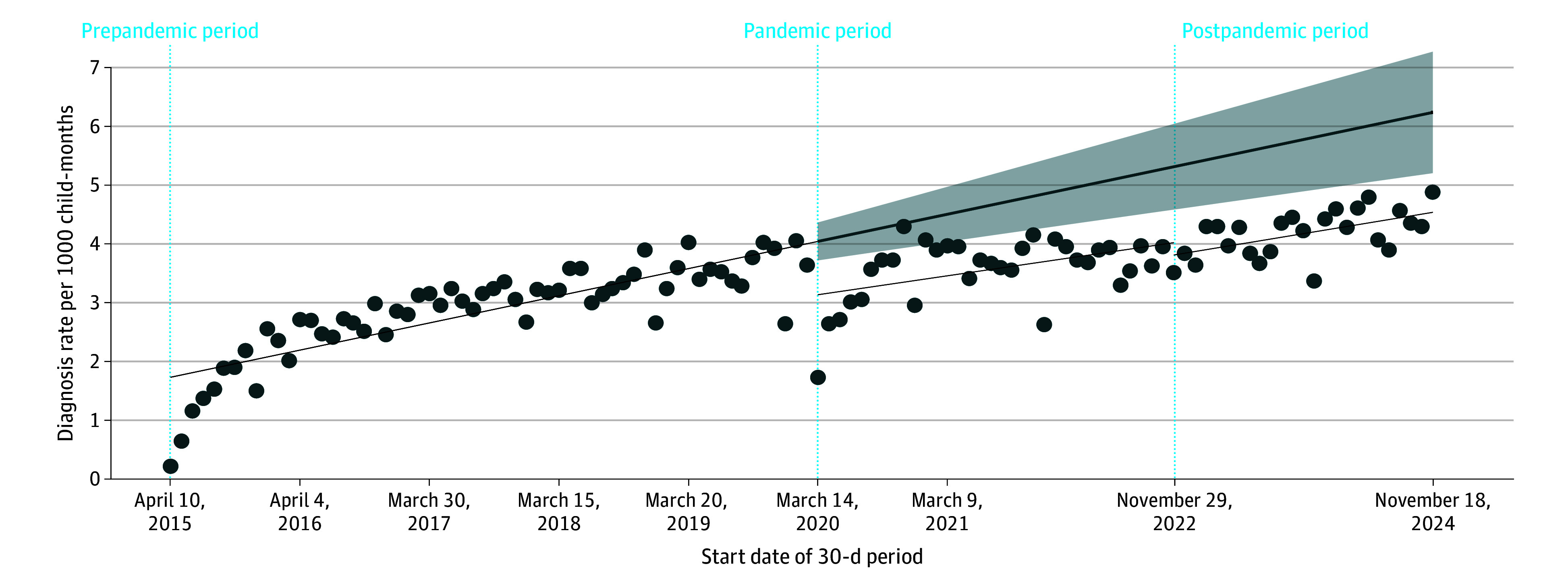
Line Graph of Observed Rates and Estimated Trends in Neurodevelopmental Diagnoses Throughout the Prepandemic, Pandemic, and Postpandemic Periods Shaded areas indicate 95% CIs.

Because we defined a new developmental diagnosis as 2 consecutive visits at least 30 days apart, rates at the onset of the prepandemic period were low (only 25.0% of diagnoses [32 605 of 130 418] were made within 2.4 months of the initial visit vs 81.5% [106 339 of 130 418] by 12 months) and appeared as outliers when compared with subsequent rates. For sensitivity analysis, we added an interruption on the 13th month of the prepandemic period to model the gradual increase in diagnoses and to accommodate the initial outliers. We also added an interruption on the month after the first-year anniversary of the pandemic (March 9 to April 7, 2021). The modified ITSA model provided additional information; however, the conclusion remained consistent with those of the original analysis (eTable 2 in Supplement 1). There was a rapid 12-month rate recovery (slope = 0.17; 95% CI, 0.11-0.22; *P* < .001) following the marked decrease after the pandemic was declared on March 14, 2020 (change in rate level, −1.38; 95% CI, −1.69 to −1.08; *P* < .001). Rates then stabilized after the 1-year anniversary of the pandemic, remaining steady until the end of the pandemic period on November 28, 2022 (slope = 0.001; 95% CI, −0.01 to 0.01; *P* = .86). The change in rate trends in the postpandemic period returned to prepandemic levels (change in trends, 0.01; 95% CI, −0.002 to 0.02; *P* = .11). eFigure 1 in Supplement 1 displays the observed rates, model-estimated values, and the counterfactual values.

## Discussion

This population-based cohort study of Ontario children aged 6 years or younger showed no significant differences in the rates of new neurodevelopmental diagnoses from the prepandemic rates in either the pandemic or postpandemic period. However, there was a marked decrease in the rates of new neurodevelopmental diagnoses in the first 30 days after the COVID-19 pandemic was declared. Rates recovered and remained stable for the duration of the pandemic period. Consistent with the literature,^3^ boys demonstrated higher rates of new neurodevelopmental diagnoses across all study periods. However, the same overall rate trends of new neurodevelopmental diagnoses across study periods were observed when examined by sex.

Restrictions on health care access at the onset of the pandemic were likely associated with the observed decrease in neurodevelopmental diagnoses during the first month of the pandemic period. Consistent with these findings, there was a decrease in the number of visits for new neurodevelopmental problems during the first 30 days after the pandemic was reported in Ontario.^17^ Similarly, primary care visit rates among children decreased to 80% of what was expected in Ontario and 82% of what was expected in Manitoba during the 9 months after the onset of the pandemic.^18^ In Ontario, pediatric mental health care visits also showed a marked decrease in the first month of the pandemic.^9^ In British Columbia, large numbers of adolescents reported unmet needs for mental health services during the first year of the pandemic.^19^ A review of studies from 49 countries reported a median 56% decrease in in-person health care utilization over the first 6 months of the pandemic, with diagnostic visits more impacted than visits for medical treatments.^20^

Although the overall rate of new neurodevelopmental diagnoses remained stable throughout the pandemic period, there was a marked rate recovery beginning 1 month after the onset of the pandemic that persisted over the following 11 months, followed by no appreciable change for the remainder of the period. The rapid implementation of virtual health care in Ontario may have been associated with the increase in health care access and rates of new diagnoses over that 11-month period, which then stabilized during the remainder of the pandemic period.^9^ Mental health care and developmental care are well suited to virtual service delivery. The shift to virtual care during the pandemic was especially amenable to developmental monitoring and anticipatory guidance, ensuring that parents continued to receive education and counseling to support their children’s early development during lockdowns.^21^

Mental health visit rates in Ontario also rebounded, exceeding expected levels within 3 months and remaining 10% to 15% higher than expected for the subsequent 6 months.^9^ Up to 75% of pediatric mental health visits were conducted virtually in Ontario during the pandemic.^9^ Results from the Medical Expenditure Panel Survey in the US reported that nearly one-third of child and adolescent mental health outpatients during the 2021 pandemic year received 1 or more of their visits via videoconference.^22^ US data also showed a 154% increase in telehealth visits for any presenting problem shortly after the onset of the pandemic compared with the same period in the year prior to the pandemic.^23^ Studies have shown that both families and clinicians were satisfied with virtual care and considered it an acceptable modality for children with neurodevelopmental and behavioral conditions.^24,25^ Diagnostic rates increased during the postpandemic period at a rate comparable with that observed before the pandemic. However, the rate was insufficient to make up for the initial decrease in neurodevelopmental diagnoses that occurred at the onset of the pandemic. The postpandemic period in Ontario moved from temporary pandemic billing codes to a more permanent virtual care billing structure, thereby supporting the sustained use of virtual care delivery alongside in-person care.^26^ This hybrid system of service delivery likely improved access to care in the postpandemic period. However, given the ongoing strain on the health care system and the backlogs resulting from early pandemic restrictions, health care capacity in the postpandemic period may not be able to fully compensate for the initial limitations in access during the pandemic.^27,28^

Studies directly reporting on child developmental outcomes during the pandemic demonstrated mixed results, varying by timing of exposure (prenatal exposure to SARS-CoV-2 vs postnatal exposure to the pandemic period) and by child age. Regarding studies reporting on prenatal exposure and neurodevelopmental outcomes, a meta-analysis of 2 studies did not find an association between prenatal exposure to SARS-CoV-2 and the overall risk of neurodevelopmental impairment during the first year of life; however, a small increased risk of fine motor impairment was observed at 12 months of age.^29^ A large US cohort study reported an increased risk of neurodevelopmental diagnoses at 12 months among male children but not female children.^30^ These differences were no longer statistically significant by 18 months. Other large cohort studies in the US and Europe found no associations between prenatal exposure to SARS-CoV-2 and neurodevelopmental outcomes up to 24 months of age.^31,32^ More recently, a retrospective cohort study of more than 18 000 births in the US reported a 29% higher odds of neurodevelopmental diagnosis by 3 years of age among children prenatally exposed to SARS-CoV-2.^33^ Risk was higher among male children than female children and appeared greatest among those exposed during the third trimester of pregnancy.

Regarding studies evaluating exposure to the pandemic period and risk of neurodevelopmental impairment, a meta-analysis of 2 studies found that being born or raised during the pandemic was not associated with overall risk of neuroimpairment; however, children exposed to the pandemic period were more likely to be at risk of communication impairment at 12 months compared with their prepandemic counterparts.^29^ A large US cohort study of 50 205 children reported modest decreases in developmental screening scores in the communication, problem-solving, and person-social domains among children aged 5 years or younger who were exposed to the pandemic, with no changes in fine or gross motor domains compared with prepandemic scores.^34^ Among this cohort, infants showed similar decreases in communication and problem-solving screening scores; however, no changes were observed in the other domains compared with prepandemic scores. Gestational exposure to SARS-CoV-2 was not known for this cohort.

Although our population-level findings do not indicate an increase in neurodevelopmental diagnoses after the COVID-19 pandemic, it is possible that associations observed in specific cohorts of children with prenatal exposure to SARS-CoV-2 were not detected at the population level. In addition, some children may have experienced transient or mild developmental delays during the pandemic; however, these impairments may not have been of sufficient severity or did not persist long enough to result in a formal diagnosis.

### Strengths and Limitations

A strength of this study was the use of provincial population-level data spanning 5 years before the pandemic and 4 years after the pandemic was declared. Limitations included lack of individual measures on sociodemographic characteristics of the study population; thus, we could not comment on how these factors may have influenced neurodevelopmental diagnoses. Furthermore, we did not evaluate potential interaction effects between income quintiles and the association between the pandemic period and neurodevelopmental diagnoses; therefore, we were unable to assess whether the association of the pandemic with neurodevelopmental diagnoses varied across income groups. In addition, we were not able to distinguish between in-person and virtual visits using diagnostic billing codes and do not know how the mode of health care utilization influenced neurodevelopmental diagnoses. Furthermore, the absence of clinical data limited our ability to interpret the circumstances and extent of the neurodevelopmental diagnoses identified through billing codes. Finally, our study lacked a control group; given the global nature of the COVID-19 pandemic and the widespread disruptions to health care access, an appropriate control cohort was unlikely to exist.

## Conclusions

In this population-based cohort study of Ontario children aged 6 years or younger, there was a marked decrease in neurodevelopmental diagnoses after the onset of the pandemic, followed by recovery and stabilization to prepandemic rates in the pandemic and postpandemic periods. Although our findings are reassuring, system backlogs and long wait times may obscure true neurodevelopmental diagnostic rates in the pandemic and postpandemic periods. Furthermore, the increasing baseline rates of neurodevelopmental problems underscores the need for robust longitudinal studies with extended follow-up periods to determine the long-term neurodevelopmental sequelae among children who experienced the COVID-19 pandemic and to inform planning for future pandemics.

## Notes

Supplement 1. **eTable 1.** Frequency of Each Neurodevelopmental Diagnosis by Pandemic Period **eTable 2.** Interrupted Time Series Sensitivity Analyses With Five Time Periods **eTable 3.** Interrupted Time Series Sensitivity Analyses Stratified by Sex **eFigure 1.** Sensitivity Analysis: Observed Rates and Estimated Trends in Neurodevelopmental Diagnoses Throughout the Pre-, During-, and Post-Pandemic Periods **eFigure 2.** Interrupted Time Series Sensitivity Analyses Stratified by Sex

Supplement 2.
Data Sharing Statement
