# Supplementary material for: Neurodevelopmental Diagnoses Before, During, and After the COVID-19 Pandemic
Source: JAMA Netw Open. 2026 Apr 8;9(4):e265683. doi: 10.1001/jamanetworkopen.2026.5683 (PMC13063079; doi:10.1001/jamanetworkopen.2026.5683)
Supplement: Supplement 1. — eTable 1. Frequency of Each Neurodevelopmental Diagnosis by Pandemic Period eTable 2. Interrupted Time Series Sensitivity Analyses With Five Time Periods eTable 3. Interrupted Time Series Sensitivity Analyses Stratified by Sex eFigure 1. Sensitivity Analysis: Observed Rates and Estimated Trends in Neurodevelopmental Diagnoses Throughout the Pre-, During-, and Post-Pandemic Periods eFigure 2. Interrupted Time Series Sensitivity Analyses Stratified by Sex [file jamanetwopen-e265683-s001.pdf]

Supplementary Online Content

Freeman SJ, Nisenbaum R, Sgro MD. Neurodevelopmental diagnoses before, during, and after the COVID-19 pandemic. *JAMA Netw Open.* 2026;9(4):e265683. doi:10.1001/jamanetworkopen.2026.5683

eTable 1. Frequency of Each Neurodevelopmental Diagnosis by Pandemic Period
eTable 2. Interrupted Time Series Sensitivity Analyses With Five Time Periods
eTable 3. Interrupted Time Series Sensitivity Analyses Stratified by Sex
eFigure 1. Sensitivity Analysis: Observed Rates and Estimated Trends in Neurodevelopmental Diagnoses Throughout the Pre-, During-, and Post-Pandemic Periods
eFigure 2. Interrupted Time Series Sensitivity Analyses Stratified by Sex

This supplementary material has been provided by the authors to give readers additional information about their work.

© 2026 Freeman SJ et al. *JAMA Network Open.*

eTable 1. Frequency of Each Neurodevelopmental Diagnosis by Pandemic Period

**OHIP diagnosis code, n (%)**	**Pre-pandemic** (March 14, 2015 - March 13, 2020)	**Pandemic** (March 14, 2020 - November 30, 2022)	**Post-pandemic** (December 1, 2022 - December 31, 2024)
Total Number of Children	130,418	86,383	75,095
Childhood psychosis or autism	7,163 (5.5)	5,732 (6.6)	6,092 (8.1)
Anxiety neurosis, hysteria, neurasthenia, obsessive compulsive neurosis, reactive depression	5,704 (4.4)	4,743 (5.5)	3,052 (4.1)
Personality disorders (e.g., paranoid personality, schizoid personality, obsessive compulsive personality)	69 (0.1)	47 (0.1)	54 (0.1)
Psychosomatic disturbances	76 (0.1)	74 (0.1)	29 (0.0)
Habit spasms, tics, stuttering, tension headaches, anorexia nervosa, sleep disorders, enuresis	7,054 (5.4)	5,030 (5.8)	3,599 (4.8)
Adjustment reaction	485 (0.4)	294 (0.3)	168 (0.2)
Behaviour disorders of childhood and adolescence	23,538 (18.0)	18,824 (21.8)	15,161 (20.2)
Hyperkinetic syndrome of childhood	1,912 (1.5)	1,255 (1.5)	1,255 (1.7)
Specified delays in development (e.g., dyslexia, dyslalia, motor retardation)	45,108 (34.6)	34,726 (40.2)	25,781 (34.3)
Mental retardation	407 (0.3)	324 (0.4)	321 (0.4)
Other cerebral degenerations	163 (0.1)	198 (0.2)	120 (0.2)
Cerebral palsy	636 (0.5)	309 (0.4)	261 (0.3)
Other diseases of central nervous system (e.g., brain abscess, narcolepsy, motor neuron disease, syringomyelia)	3,463 (2.7)	1,499 (1.7)	1,145 (1.5)
Trigeminal neuralgia, tic douloureux	31 (0.0)	14 (0.0)	10 (0.0)
Amblyopia, visual field defects	15,911 (12.2)	5,545 (6.4)	9,533 (12.7)
Blindness and low vision	154 (0.1)	105 (0.1)	59 (0.1)
Deafness	13,618 (10.4)	4,338 (5.0)	6,162 (8.2)
Parent-child problems (e.g., child-abuse, battered child, child neglect)	1,318 (1.0)	595 (0.7)	374 (0.5)
Educational problems	3,123 (2.4)	2,342 (2.7)	1,583 (2.1)
Social maladjustment	99 (0.1)	129 (0.1)	141 (0.2)
Other problems of social adjustment	386 (0.3)	260 (0.3)	195 (0.3)

© 2026 Freeman SJ et al. *JAMA Network Open.*

eTable 2. Interrupted Time Series Sensitivity Analyses With Five Time Periods

Period*	Parameter	Estimate (95% CI)	p-value
Pre-pandemic 1	Initial rate level	0.70 (0.39 - 1.00)	<0.001
	Slope	0.17 (0.12 - 0.22)	<0.001
Pre-pandemic 2	Change in rate level (pre-pandemic 2 minus pre-pandemic 1)	-0.06 (-0.36 to 0.25)	0.72
	Change in rate trends (pre-pandemic 2 minus pre-pandemic 1)	-0.14 (-0.19 to -0.09)	<0.001
	Slope	0.02 (0.02 - 0.03)	<0.001
Pandemic 1	Change in the rate level (pandemic 1 minus pre-pandemic 2)	-1.38 (-1.69 to -1.08)	<0.001
	Change in rate trends (pandemic 1 minus pre-pandemic 2)	0.14 (0.08 - 0.20)	<0.001
	Slope	0.17 (0.11 - 0.22)	<0.001
Pandemic 2	Change in the rate level (pandemic 2 minus pandemic 1)	-0.63 (-1.05 to -0.21)	0.004
	Change in rate trends (pandemic 2 minus pandemic 1)	-0.16 (-0.22 to -0.11)	<0.001
	Slope	0.001 (-0.01 to 0.01)	0.86
Post-pandemic	Change in rate level (post-pandemic minus pandemic 2)	0.08 (-0.07 to 0.23)	0.31
	Change in rate trends (post-pandemic minus pandemic 2)	0.03 (0.02 - 0.04)	<0.001
	Change in rate trends (post pandemic minus pre-pandemic 2)	0.01 (-0.002 to 0.02)	0.11
	Slope	0.03 (0.02 - 0.04)	<0.001

*Pre-pandemic 1: April 10, 2015 to April 3, 2016; Pre-pandemic 2: April 4, 2016 to March 13, 2020; Pandemic 1: March 14, 2020 to March 8, 2021; Pandemic 2: March 9, 2021 to November 28, 2022; Post-pandemic: November 29, 2022 to December 17, 2024
CI: Confidence Interval

© 2026 Freeman SJ et al. *JAMA Network Open.*

eTable 3. Interrupted Time Series Sensitivity Analyses Stratified by Sex

		Males		Females	
**Period***	**Parameter**	**Estimate (95% CI)**	**p-value**	**Estimate (95% CI)**	**p-value**
Pre-pandemic	Rate level at beginning of the pre-pandemic period	2.08 (1.48 - 2.69)	<0.001	1.37 (0.98 – 1.77)	<0.001
	Pre-pandemic slope	0.05 (0.03 - 0.06)	<0.001	0.03 (0.02 - 0.04)	<0.001
Pandemic	Change in the rate level that occurred immediately after the start of the pandemic period compared with the counterfactual pre-pandemic	-1.13 (-1.96 to -0.31)	0.01	-0.68 (-1.16 to -0.20)	0.01
	Change in rate trends or difference between during-pandemic and pre-pandemic slopes	-0.01 (-0.05 to 0.03)	0.57	-0.01 (-0.04 to 0.01)	0.37
	Pandemic slope	0.04 (-0.0003 to 0.07)	0.05	0.02 (-0.003 to 0.04)	0.09
Post-pandemic	Change in rate level that occurs immediately after the start of the post-pandemic period compared with the counterfactual during the pandemic period	-0.28 (-0.76 to 0.20)	0.25	-0.14 (-0.44 to 0.17)	0.39
	Change in rate trend or difference between post-pandemic and during-pandemic slopes	-0.01 (-0.05 to 0.03)	0.70	0.01 (-0.01 to 0.04)	0.25
	Change in rate trend or difference between post-pandemic and pre-pandemic slopes	-0.02 (-0.04 to 0.002)	0.08	0.003 (-0.01 to 0.02)	0.70
	Post-pandemic slope	0.03 (0.01 - 0.04)	<0.001	0.03 (0.02 to 0.05)	<0.001

*Pre-pandemic 1: April 10, 2015 to April 3, 2016; Pre-pandemic 2: April 4, 2016 to March 13, 2020; Pandemic 1: March 14, 2020 to March 8, 2021; Pandemic 2: March 9, 2021 to November 28, 2022; Post-pandemic: November 29, 2022 to December 17, 2024
CI: Confidence Interval

© 2026 Freeman SJ et al. *JAMA Network Open.*

eFigure 1. Sensitivity Analysis: Observed Rates and Estimated Trends in Neurodevelopmental Diagnoses Throughout the Pre-, During-, and Post-Pandemic Periods

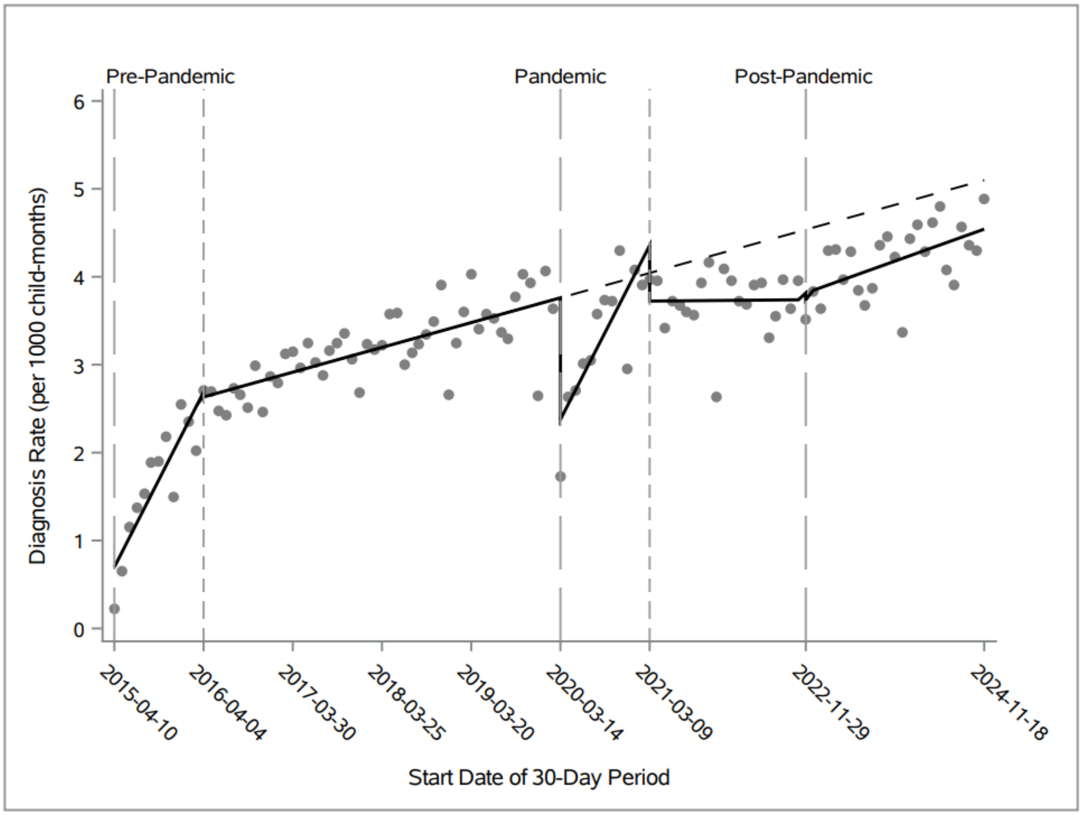

© 2026 Freeman SJ et al. *JAMA Network Open.*

eFigure 2. Interrupted Time Series Sensitivity Analyses Stratified by Sex

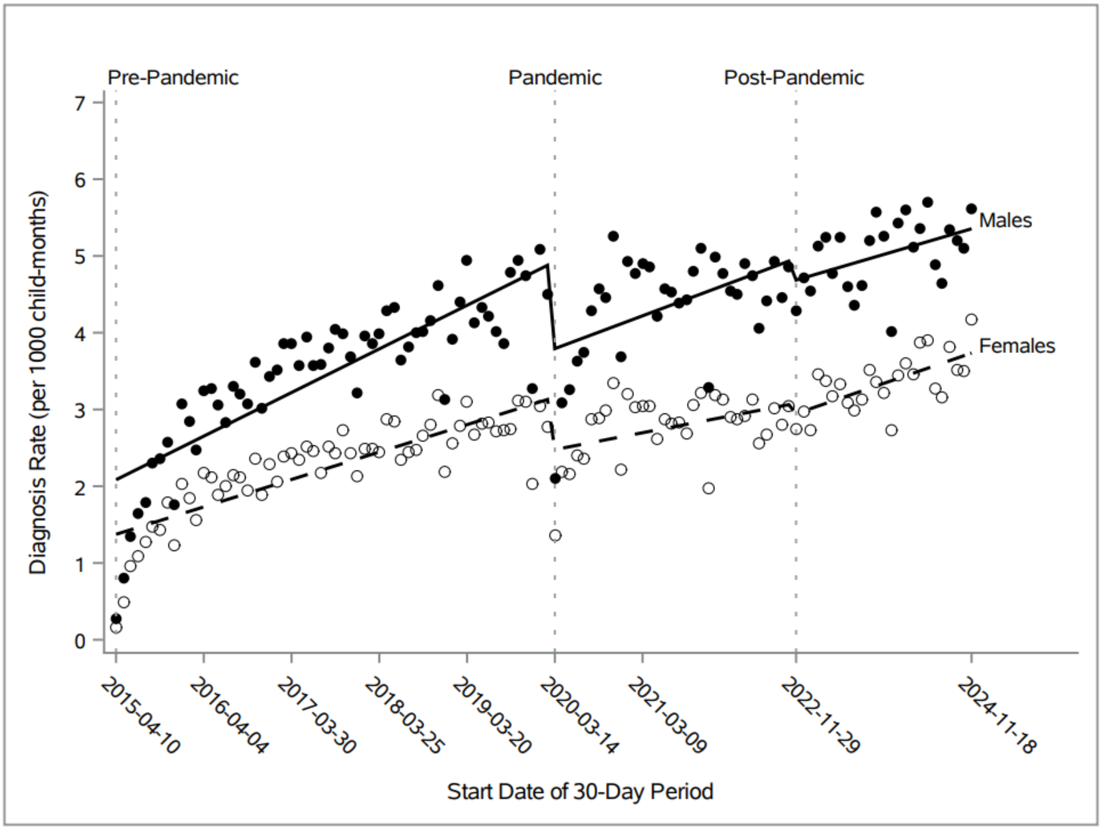

© 2026 Freeman SJ et al. *JAMA Network Open.*
