# Supplementary material for: Neurodevelopmental Diagnoses Before, During, and After the COVID-19 Pandemic
Source: JAMA Netw Open. 2026 Apr 8;9(4):e265683. doi: 10.1001/jamanetworkopen.2026.5683 (PMC13063079; doi:10.1001/jamanetworkopen.2026.5683)
Supplement: Supplement 2. — Data Sharing Statement [file jamanetwopen-e265683-s002.pdf]

Data Sharing Statement

Freeman. Neurodevelopmental Diagnoses Before, During, and After the COVID-19 Pandemic. *JAMA Netw Open*. Published April 08, 2026. doi:10.1001/jamanetworkopen.2026.5683

Data
Data available: Yes
Data types: Data dictionary
How to access data: Please send any data requests to sloane.freeman@unityhealth.to
When available: With publication

Supporting Documents
Document types: None

Additional Information
Who can access the data: Researchers whose proposed use of the data has been approved.
Types of analyses: For any purpose that has been approved.
Mechanisms of data availability: The data will be made available wiht investigator support, after approval of a proposal and with a signed data access agreement.
